# Hydrolysis and oxidation of protein and lipids in dry-salted grass carp (*Ctenopharyngodon idella*) as affected by partial substitution of NaCl with KCl and amino acids

**DOI:** 10.1039/c9ra07019b

**Published:** 2019-12-02

**Authors:** Xiuyun Guo, Shanan Chen, Jiayue Cao, Jingying Zhou, Yanzheng Chen, Muneer Ahmed Jamali, Yawei Zhang

**Affiliations:** College of Food Science and Technology, National Center of Meat Quality and Safety Control, Nanjing Agricultural University Nanjing 210095 China zhangyawei@njau.edu.cn; Synergetic Innovation Center of Food Safety and Nutrition Nanjing China; Department of Animal Products Technology, Sindh Agriculture University Tandojam Pakistan

## Abstract

To obtain healthier meat products with reduced Na content, the salt substitute containing l-histidine and l-lysine was compared with NaCl in the hydrolysis and oxidation of protein and lipids of dry-salted fish during processing. Compared with NaCl-treated fish (S-F), salt substitute treated fish (SS-F) had a lower Na content, higher moisture content and lower hardness. Sensory analysis showed that salt substitute didn't affect the acceptability of salted fish. The free fatty acids of SS-F treated fish had a slight tendency toward lipolysis at the end of processing. Additionally, the conjugated diene value, lipoxygenase activity and malondialdehyde value were lower in the ventral and dorsal muscles for the SS-F treatment. Meanwhile, the protein carbonyls and thiol groups were significantly decreased as cathepsin B and L activities and FAA content were increased in the ventral and dorsal muscles for the SS-F treatment. l-Histidine and l-lysine accelerated the hydrolysis (inhibit the oxidation) of protein and lipids in dry-salted grass carp, illustrating that l-histidine and l-lysine will be a positive approach to develop healthier meat products.

## Abstract 2

To obtain healthier meat products with reduced Na content, the salt substitute containing l-histidine and l-lysine was compared with NaCl in the hydrolysis and oxidation of protein and lipids of dry-salted fish during processing.

## Graphical abstract

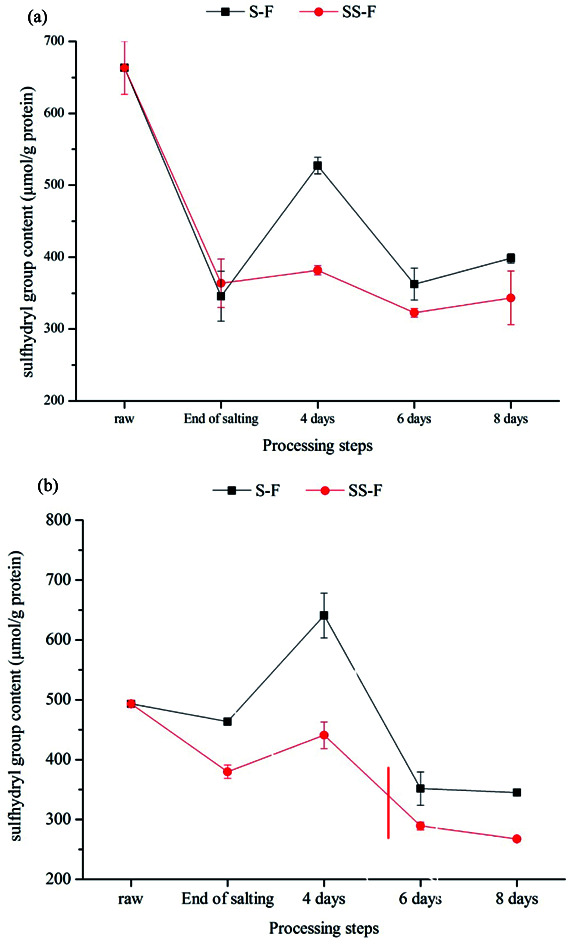

## Introduction

Sodium chloride (NaCl) is generally used in fish processing because it acts as preservative and has several other positive technological properties.^1,2^ Traditional aquatic products with 1.0–11.6% NaCl (w/w) or high sodium content is a well-known characteristic of dry-salted fish products.^3,4^ On the other hand, increased consumption of sodium (Na) is linked with the risk of high blood pressure and the development of some other cardiovascular diseases.^5,6^ Therefore, approaches towards reducing the NaCl content of salted fish has attracted attention throughout the world.

Salted fish was easy to preserve as well as it has a typical developed flavor due to hydrolysis and oxidation of protein and lipid and the rate of hydrolysis and oxidation of protein and lipid vary according to the composition of salt used during the processing of salted fish.^7^ On one hand, lipid and protein oxidation occurring during the processing is an important factor for quality deterioration, such as deterioration of the texture, nutritional value, discoloration of salted fish.^8,9^ On the other hand, lipid and protein hydrolysis affect sensory and nutritional qualities of dry-cured meat. The important factors such as salt type and content affected proteolysis and lipolysis which were important biochemical changes during processing, leading to the development of the desired flavor, texture and color in the dry-cured meat products.^10^ Proteolysis and lipolysis has been attributed to endogenous enzymes.^11,12^

In previous studies, most of the researchers focused on the applications of KCl combined with MgCl_2_ or CaCl_2_ in salted fish.^13–15^ However, magnesium salts and calcium hindered the proper penetration of salt into fish tissues^16,17^ and the availability of KCl (more than 50%) might attenuate the flavor and produce bitterness.^12,18^ Therefore, considerable interest had been focused on the application of amino acids as a salt substitute component for meat products, and it was found that l-arginine or l-lysine (Lys) significantly improved the water holding capacity, a* value and textural properties of pork sausages.^19^ A salt substitute containing Lys was also reported to result in good physicochemical properties of surimi gels.^20,21^ In our previous study, the application of l-histidine (His) and Lys as salt substitutes remarkably reduced the TBARS value of dry-cured loin compared to the NaCl treatment.^22^ However, based on our knowledge, research regarding use of amino acids as salt substitute components in dry-salted fish has not been previously reported.

Therefore, in the present study, grass carp (*Ctenopharyngodon idella*), a freshwater herbivorous fish that generally found in the rivers draining East Asia and was cultured around the globe,^23^ was used for the experiment and salted during processing, and the effect of amino acids (His and Lys) as salt substitute on the hydrolysis and oxidation of protein and lipid in salted fish was studied. As well known, in the dorsal muscle the value of lipids is remarkably lower compared to the ventral muscle in fish, such as gilthead sea bream, rainbow trout and European sea bass.^24^ For that reason, there was a need to study and compare the ventral and dorsal muscles of fish for lipid oxidation and hydrolysis, particularly under low NaCl circumstances. Moreover, interactions among the components His and Lys might have a role in salty taste of NaCl.^25^ Therefore, the current study was designed to analyze the influence of NaCl (salt) and Lys and His (salt substitute) on hydrolysis and oxidation of protein and lipid in the ventral and dorsal muscles of dry-salted grass carp (*Ctenopharyngodon idella*) throughout the processing.

## Materials and methods

### Fish sample preparation

Grass carp (*Ctenopharyngodon idella*) weighing about 1.5 ± 0.5 kg was purchased from the market of Nanjing, China in September 2017. The live fishes for the samples were collected in oxygen aerated water and transported within 20 min to the laboratory in the College of Food Science and Technology, Nanjing Agricultural University, China. After being kept for 1 h at ambient temperature, the collected fishes were washed, headed, eviscerated and then drained at 4–10 °C for 15 min. Three fishes were randomly selected and evaluated as the raw material. Next, the six fishes for each of the other processing steps were divided into a salt-fish (S-F) group and a salt-substitute-fish (SS-F) group, with three fishes in each group. The salt substitute purchased from Rolys Co., Ltd, Nanjing, China contained 51.3 g/100 g of KCl, 39.7 g/100 g of NaCl, 7 g/100 g l-lys and 2 g/100 g l-his. For these two groups, the samples were salted with 4% NaCl (S-F) or salt substitute (SS-F) at 4 °C for 48 h. After salting, samples were dried at 15 °C for 24 h at 75% relative humidity (RH). Then, samples were dried at 22 °C for 24 h at 70% RH and holding at 22 °C for 24 h at 65% RH; holding at 22 °C for 24 h at 65% RH; samples were dried at 24 °C for 24 h at 65% RH and holding at 24 °C for 24 h at 60% RH; finally, samples were dried at 26 °C for 24 h at 55% RH and holding at 26 °C for 24 h at 50% RH. At the 4 processing points (end of 2 days salting, drying-processing 2, 4, and 6 days) the ventral and dorsal muscles of the fishes were trimmed off, skinned and collected to obtain the samples for further use. All the sampled muscles were packed using vacuum and kept at −20 °C before use.

### Physicochemical characteristics

The potassium and sodium content of the samples were analyzed as described by Zhang.^22^ HCl (0.3 M) was used to prepare a solution using deionized distilled water to wash the glassware for overnight before use. The moisture content was determined using oven (ISO Norm R-1442, 1979) drying method at 100 °C until constant weight. Extraction of total lipids was performed by homogenization of minced muscle (5 g) in 30 ml of chloroform/methanol (2 : 1, v/v) as reported by Folch.^26^ All the analysis was performed in triplicate.

The color of the samples was determined according to the methods described by Cui.^27^ The samples were measured on a freshly cut upper surface of dry-cured beef using a CR-400 colorimeter (Minolta, Osaka, Japan). Before each series of measurements, the instrument was calibrated using a white ceramic tile. Color of the surface was acquired and measured three times for each sample to arrive at the final averaged value. The values are expressed and represented as lightness (L*), redness (a*) and yellowness (b*).

Texture profile analysis (TPA) was carried out by compression to 30% with a compression probe (P50) of 19.85 cm^2^ surface contact using a texture analyser TAXT2i (Stable Micro Systems, Godalming, UK). Other parameters were as follows: pre-test speed, 1.0 mm s^−1^; post-test speed, 1.0 mm s^−1^; time between two compressions, 5.0 s. Hardness (N), springiness (mm), chewiness (mJ) and cohesiveness (N) were recorded.

### Sensory analysis

The samples were steamed with the boiled water for 15 min before they were subjected to sensory analysis to determine the influence of the salt substitution on sensory properties. A panel was conducted with thirty panellists selected from the National Center of Meat Quality and Safety Control of China. The panellists were trained according to the methodology proposed by ISO regulations (ISO 8586:2012) during two weeks with the attributes and the scale to be used. Quantitative descriptive analyses^28^ were carried out to assess the intensity color, saltiness, umami, off-flavor and overall acceptability of the finished product. The samples were presented to the panelists with three-digit codes and in random order. The intensity of every attribute was expressed on an unstructured scale from 0 (sensation not perceived) to 10 (maximum of the sensation). During sensory evaluation, the panellists were situated in private cabinet illuminated with red light. Water to clean the palates and remove residual flavors was used at the beginning of the session and in between samples. The final scores were averaged over all panelists.

### Lipases activities assay

The total lipase activity was carried out using a Total Lipase Test kit (A054-2, Nanjing Jiancheng Bioengineering Co Ltd.). The activities of neutral lipase and acid lipase were determined using the previously documented method by Vestergaard.^29^ The activity of phospholipase was analyzed as reported by Toldrá.^30^ The activities of enzymes were presented as nmol of released 4-methylumbelliferone per h per g protein. The analysis was performed in triplicate.

### Analysis of total lipids and free fatty acids

Total lipids were extracted from 5 g of minced muscles according to the method described by Folch, Lees, and Sloane-Stanley^26^ using 25 ml of chloroform : methanol (2 : 1) as the solvent. The extracted lipid was expressed as mg g^−1^ dry matter. One hundred milligrams of the dry extracted lipids was separated using NH_2_-aminopropyl minicolumns, following the method of Kaluzny, Duncan, Merritt, and Epps.^31^ Free fatty acids (FFAs) were eluted with 3.0 ml of diethyl ether: acetic acid 2%. The amounts of FFAs was expressed as mg g^−1^ total lipids. Fatty acids composition was analyzed using gas–liquid chromatography. Gas chromatograph (Trace GC Ultra, Thermo Electron Corporation, Waltham, USA) was used to analyze the methyl esters of fatty acids as reported by Morrison and Smith.^32^

### Measurement of conjugated diene

The conjugated diene and carbonyl value were analyzed using the procedure reported by Coutron-Gambotti and Gandemer^33^ with slight modifications. Lipid sample (10 mg) was mixed with 8 ml of cyclohexane and the absorbance was read at 215 and 232 nm. Finally, the absorbance at 232 nm was divided with the absorbance taken at 215 nm to calculate the conjugated diene. All the samples were analyzed in triplicate.

### Lipoxygenase (LOX) activity determination

The LOX activity was determined using a LOX ELISA kit (Jiancheng, Nanjing, China). Minced sample (2 gm) was homogenized on ice using 18 ml of PBS (pH = 7.4, 0.01 M). Thereafter, homogenate was centrifuged (5000*g*, for 10 min) at 4 °C to get the supernatant. Fifty microliters of sample or standard was poured into the wells; afterward, 100 μl of enzyme was added to conjugate to sample wells and standard wells excluding the blank well. The wells were roofed with an adhesive strip and incubated at 37 °C for 60 min. Next, substrate A and B (50 μl) were added to each well, after careful mixing incubated for 15 min at 37 °C. Lastly, in each well stop solution (50 μl) was added and the activity of LOX was measured at 450 nm. Triplicate analysis was performed for the samples.

### Measurement of TBARS

The TBARS value in the samples was analyzed using the procedure reported by Salih, Smith, Price, and Dawson^34^ and the value was presented as mg of malondialdehyde (MDA) per kg of muscle. Triplicate analysis was performed for the samples.

### Protein carbonyl measurements

Protein oxidation, as measured by the total carbonyl content, was evaluated by derivatisation with DNPH according to the method described by Ollilainen.^35^ Samples were thawed, minced and then homogenized 1 : 10 (w/v) in pyrophosphate buffer (pH 7.4) (PB) consisting of 2 mM Na_4_P_2_O_7_, 10 mM Tris–maleate, 100 mM KCl, 2 mM MgCl_2_ and 2 mM EGTA using an ultraturrax homogenizer for 30 s. The homogenates were divided in two equal aliquots of 0.1 mL. Then, proteins were precipitated in both aliquots by adding 1 mL of 10% TCA and centrifuged for 5 min at 5000 rpm. Finally, the supernatants were removed and one pellet was treated with 1 mL 2 M HCl and the other one with an equal volume of 0.2% (w/v) DNPH in 2 M HCl. Both samples were incubated for 1 h at room temperature (shaken every 15 min). After that, samples were precipitated with 1 mL of 10% TCA and washed twice with 1 mL of 1 : 1 ethanol/ethyl acetate (v/v), shaken, and centrifuged for 5 min at 10 000 rpm. The pellets were then dissolved in 1.5 mL of 20 mM sodium phosphate buffer pH 6.5 containing 6 M guanidine hydrochloride, stirred and centrifuged for 2 min at 5000 rpm to remove insoluble fragments. Protein concentration was calculated from absorption at 280 nm using BSA as standard. The amount of carbonyls was measured at 370 nm and expressed as nmol of carbonyl per mg of protein using the adsorption coefficient for the protein hydrazones (21.0 mM^−1^ cm^−1^).

### Sulphydryl (SH) content

The SH group levels were determined according to the method of Cui.^36^ 0.5 ml of fish sample homegenate was mixed with 2.5 mL of Tris-Gly-8 M urea and 0.02 mL of 4 mg mL^−1^ 5,5′-dithiobis-2,2′-nitrobenzoic acid (DTNB). After incubation at 25 °C for 30 min, the absorbance at 412 nm (*A*_412_) was recorded. The SH group level was calculated by eqn (1).

1 SH group level (μmol per g per fish proteins) = 73.53*A*_412_ × *D*/*C*

where *D* is the dilution coefficient (6.04); *C* (mg mL^−1^) is the protein concentration in tested sample.

### Assay of enzyme activities

The extracts containing cathepsin B (CTS B, EC 3.4.22.1) and L (CTS L, EC 3.4.22.15) were obtained from 5 g samples using the methods described by Armenteros *et al.* (2012).^5^ The activity of enzyme was measured by fluorometric assays using aminoacyl-7-amido-4-methyl coumarin (aa-AMC) (Sigma-Aldrich Co., St. Louis, MO) as fluorescent substrates. Activity levels of cathepsin B (EC 3.4.22.1) and L (EC 3.4.22.15) are determined at pH 6.0 using 0.05 mM N-CBZ-Arg-Arg-AMC and 0.05 mM N-CBZ-Phe-Arg-AMC, respectively.

### Free amino acids (FAA) analysis

Samples for free amino acid analysis were prepared according to the procedures described by Aro^37^ with some modification. Samples (5 g) were homogenised for 1 min with 45 ml of distilled water. The homogenate was centrifuged for 20 min at 10 000*g* at 4 °C. The supernatant was filtered through filter paper and the samples were diluted 1 : 1 with 4% trichloroacetic acid (TCA) to give a final concentration of 2%. They were then incubated at 37 °C for 30 min. The supernatant was filtered again with filter paper. Thereafter, the solution was ultrafiltered through a Millipore filter having a pore diameter of 0.45 μm. Samples were analysed using a fully automated amino acid analyser HITACHI L-8900 (Hitachi Ltd., Japan).

### Statistical analysis

Data was tabulated and analysis of variance (ANOVA) was applied. Furthermore, to measure the statistical differences between the groups Duncan's multiple-range test was applied at *P* < 0.05 using SAS 8.2 (SAS Institute Inc., Cary, NC, USA). Correlations between variables were determined by correlation analyses using the Pearson's linear correlation by IBM SPSS Statistics 20 software (IBM, Chicago, IL, USA).

## Results and discussion

### Sodium, potassium and moisture contents

During the processing, in the salt substitute-treated fish (SS-F) the Na content was comparatively (*P* < 0.05) lower than that in the S-F for both ventral and dorsal muscles (Table 1). In the final dorsal muscle, the content of Na in SS-F was 52.85% less than that in the S-F group. A similar result was obtained in the ventral muscle and the decrease in Na content was 55.75%. On the contrary, a remarkable (*P* < 0.05) increase in the amount of K was found in both ventral and dorsal muscles but the K content was not more than 300 mg/100 g muscle in any sample. According to the dietary reference intakes from the Institute of Medicine, an adequate intake of K is 4700 mg per day.^38^ If the salt substitute was used to manufacture salted fish and the serving size was 100 g, which indicates that such salted fish product only contributes about one tenth of the 4700 mg per day adequate intake.

**Table 1:** Sodium, potassium (% dry matter) and moisture content (g/100 g muscle) in dorsal and ventral muscles of salted fish during processing^a^

		Processing steps								
		Raw	End of salting		4 days		6 days		8 days	
			S-F	SS-F	S-F	SS-F	S-F	SS-F	S-F	SS-F
Dorsal muscle	Sodium	0.12 ± 0.01eA	4.92 ± 0.07bB	1.79 ± 0.14dA	5.22 ± 0.21aB	2.36 ± 0.21cA	5.39 ± 0.04aB	2.53 ± 0.05cB	5.43 ± 0.15aB	2.56 ± 0.01cB
	Potassium	2.01 ± 0.03bA	2.03 ± 0.01bA	5.26 ± 0.48aA	2.09 ± 0.05bA	5.28 ± 0.14aA	2.06 ± 0.02bA	5.43 ± 0.29aA	2.01 ± 0.08bA	5.04 ± 0.11aA
	Moisture	79.66 ± 0.74aA	77.68 ± 0.35bA	76.91 ± 0.65bA	70.79 ± 0.76dA	73.66 ± 0.33cA	66.80 ± 0.66eA	70.38 ± 0.41dA	50.84 ± 0.66gA	55.95 ± 0.37fA
Ventral muscle	Sodium	0.13 ± 0.01gA	5.61 ± 0.19cA	1.77 ± 0.09fA	5.91 ± 0.07bA	2.38 ± 0.11eA	5.96 ± 0.16abA	2.65 ± 0.04dA	6.17 ± 0.01aA	2.73 ± 0.05dA
	Potassium	1.83 ± 0.11dA	1.68 ± 0.14dA	5.01 ± 0.07cA	1.65 ± 0.08dB	5.43 ± 0.16bA	1.66 ± 0.12dB	5.93 ± 0.09aA	1.67 ± 0.15dA	5.02 ± 0.01aAB
	Moisture	79.04 ± 1.69aA	73.74 ± 0.87cB	76.43 ± 0.18bA	69.15 ± 0.51dA	71.82 ± 0.49dB	61.09 ± 0.93eB	55.21 ± 0.61fB	34.43 ± 0.52hB	40.47 ± 0.32gB

a Different capital letters in the same column indicate significant differences between the dorsal and ventral muscles for a given indicator (*P* < 0.05). Different lower-case letters in the same row indicate significant differences during the processing (*P* < 0.05).

The moisture content was decreased with the processing time and it was higher in the dorsal muscle when compared to the ventral muscle and these results are agreed with Testi,^39^ who reported that compared to the ventral muscle the percentage of moisture was higher in the dorsal muscles of rainbow trout, sea bream and sea bass. In our study at the end of drying 8^th^ day, the moisture content was 10.05% higher in dorsal muscle and 14.92% higher (*P* < 0.05) in ventral muscle of SS-F group compared to the S-F muscles, respectively. In the Armenteros's^5^ studies, they found that the moisture content of dry-cured ham was not significantly influenced by partial replacement of NaCl by KCl. Therefore, the higher moisture content might be attributed to the presence of His/Lys. These findings are in agreement with the previous studies. In Zhang's study,^40^ they implied that the presence of His/Lys reduced the cook loss and enhanced the WHC of porcine myosin gels due to exposure of hydrophobic groups of myosin through the electrostatic effect between Lys/His and myosin. In Zhou's^19^ study, they suggested that incorporation of Lys significantly enhance the pH value of pork sausage resulting in an increase in WHC, which might be due to the formation of complexes with Lys with either Ca^2+^ or Mg^2+^ endogenous metallic ions contributing to dissociation of actomyosin.^41^

### Color and texture profile analysis

The evolution of color parameters throughout the processing was shown in Table 2. At the end of processing, no significant difference (*P* > 0.05) of a* and b* between S-F and SS-F was observed in both dorsal and ventral muscle. Nevertheless, compared with S-F, the L* value of SS-F was decreased by 2.85% and 1.5% in dorsal and ventral muscle, respectively. Aliño^42,43^ found that color parameters of dry-cured loins was not affected by the partial replacement of NaCl by KCl. Therefore, the decrease of L* value might be due to the presence of His/Lys, and this was in agreement with Zhou's^19^ study, who found that the incorporation of Lys decreased the L* value of pork sausage. In the previous studies,^44,45^ it was noted that the loss of water can reduce light scattering, which could lead to loss of transparency, thus increasing the L* value. That is, the increase of moisture content could lead to the decrease of L* value. The negative correlation (Table 11) between moisture content and L* value (*r* = −0.995, *p* < 0.01) in the present study seemed to support this hypothesis. As shown above, Lys/His caused an increase of moisture content, thus decreasing L* value.

**Table 2:** Changes in color in both dorsal muscle and ventral muscle of salted fish with salt or SS treatments during processing^a^

		Processing steps								
		Raw	End of salting		4 days		6 days		8 days	
			S-F	SS-F	S-F	SS-F	S-F	SS-F	S-F	SS-F
Dorsal muscle	L*	62.45 ± 1.65a	49.23 ± 0.39b	43.63 ± 0.84c	44.01 ± 0.43c	41.01 ± 0.7d	35.48 ± 0.64e	35.60 ± 0.12e	36.39 ± 0.25e	33.85 ± 0.02f
	a*	2.42 ± 0.15a	0.86 ± 0.32d	0.84 ± 0.03d	1.23 ± 0.15cd	1.44 ± 0.18c	1.36 ± 0.20c	1.57 ± 0.21bc	2.49 ± 0.14a	2.69 ± 0.19a
	b*	1.63 ± 0.15c	2.11 ± 0.19c	5.46 ± 0.34a	5.67 ± 0.34a	4.59 ± 0.11a	5.22 ± 0.23a	5.69 ± 0.41a	5.24 ± 0.18a	5.40 ± 0.22a
Ventral muscle	L*	69.65 ± 1.17a	46.89 ± 1.03b	44.83 ± 0.36c	41.84 ± 0.51d	46.82 ± 1.08b	39.78 ± 0.74e	35.09 ± 0.57g	36.98 ± 0.25f	35.48 ± 0.11g
	a*	4.95 ± 0.02a	1.53 ± 0.18b	0.91 ± 0.14c	1.75 ± 0.35b	1.20 ± 0.36e	1.37 ± 0.25bc	1.67 ± 0.26b	1.32 ± 0.08c	1.15 ± 0.2c
	b*	3.93 ± 0.46a	3.51 ± 0.22ab	3.89 ± 0.12a	3.03 ± 0.32b	0.99 ± 0.06c	3.88 ± 0.12a	1.55 ± 0.2b	3.60 ± 0.49a	3.62 ± 0.12a

a Different lower-case letters in the same row indicate significant differ ences during the processing (*P* < 0.05).

Table 3 showed the mean values for hardness, springiness, cohesiveness and chewiness in both dorsal and ventral muscle. At the end of processing, no significant difference (*P* > 0.05) of springiness, cohesiveness and chewiness between S-F and SS-F was observed in both dorsal and ventral muscle, while a significant decrease (*P* < 0.05) of hardness in SS-F was observed both in dorsal and ventral muscle. Aliño^42^ found that the hardness of dry-cured loins was not affected by the 50% replacement of NaCl by KCl. Therefore, the decrease of hardness might be due to Lys/His. Texture traits were affected by proteolysis which was due to the effect of enzyme activity changing the protein structures.^46^ The presence of Lys/His increased the activity of cathepsin B and L (shown in Fig. 2), accelerating the hydrolysis of muscle protein, thus resulting in the decreased hardness. In fact, hardness showed a positive correlation with cathepsin B activity (*r* = 0.899, *p* < 0.05). This was in accordance with previous studies, illustrating that the higher activity of cathepsin B was involved in protein degradation which could play a crucial role in the excessive softness defects of dry-cured meat products.^5,47^

**Table 3:** Changes in texture in both dorsal muscle and ventral muscle of salted fish with salt or SS treatments during processing^a^

		Processing steps								
		Raw	End of salting		4 days		6 days		8 days	
			S-F	SS-F	S-F	SS-F	S-F	SS-F	S-F	SS-F
Dorsal muscle	Hardness (N)	11.33 ± 2.25g	22.05 ± 2.16f	20.78 ± 1.62f	29.85 ± 1.17cd	25.75 ± 1.46e	32.18 ± 2.42bc	27.57 ± 2.12de	37.42 ± 1.18a	33.29 ± 1.09b
	Springiness (mm)	1.63 ± 0.23d	2.41 ± 0.23c	3.06 ± 0.41ab	2.79 ± 0.18bc	3.21 ± 0.18a	3.22 ± 0.08a	2.69 ± 0.15bc	2.55 ± 0.13c	2.60 ± 0.30c
	Cohesiveness (N)	8.48 ± 1.94c	17.22 ± 4.08b	17.81 ± 3.27b	16.55 ± 0.89b	20.98 ± 2.10b	17.85 ± 1.55b	35.28 ± 4.25a	38.93 ± 5.50a	33.61 ± 1.53a
	Chewiness (mJ)	5.03 ± 0.57d	6.48 ± 0.80cd	5.78 ± 0.33cd	7.62 ± 2.66c	6.56 ± 0.96cd	15.53 ± 0.51b	13.08 ± 0.83b	15.78 ± 0.59a	14.57 ± 2.54ab
Ventral muscle	Hardness (N)	16.18 ± 2.86f	25.76 ± 2.75d	21.27 ± 1.55e	31.26 ± 0.93bc	27.48 ± 1.26d	32.68 ± 1.63c	28.45 ± 0.47d	40.38 ± 0.45a	37.80 ± 2.03b
	Springiness (mm)	1.41 ± 0.29c	1.91 ± 0.21b	2.40 ± 0.14a	2.14 ± 0.33a	2.45 ± 0.15a	2.52 ± 0.24a	2.53 ± 0.50a	1.88 ± 0.09b	1.80 ± 0.06bc
	Cohesiveness (N)	6.90 ± 1.07d	16.55 ± 3.43bc	18.41 ± 2.00cd	16.59 ± 4.83b	17.05 ± 3.18cd	24.02 ± 2.47b	17.56 ± 3.24cd	25.48 ± 1.92a	29.49 ± 2.46a
	Chewiness (mJ)	4.96 ± 0.60d	8.44 ± 0.86c	7.59 ± 0.42c	10.44 ± 3.95c	6.93 ± 1.06c	10.80 ± 2.59b	6.96 ± 0.52c	13.52 ± 0.37ab	12.57 ± 1.62a

a Different lower-case letters in the same row indicate significant differences during the processing (*P* < 0.05).

### Sensory analysis

Table 4 showed the mean scores for intensity color, saltiness, umami, bitterness and overall acceptability of steamed samples. A significant decrease in intensity color observed in SS-F compared to the S-F could be due to the higher moisture content caused by the presence of Lys/His both in dorsal and ventral muscle (Table 1). No significant differences were observed between S-F and SS-F with respect to saltiness in both dorsal annessd ventral muscle. Regarding saltiness, the assessors didn't notice the different salty taste between S-F and SS-F. This could be due to the fact that salt substitute containing Lys/His had the similar salty taste with NaCl, since His/Lys might contribute to the salty taste of NaCl through the interactions.^25^ Notably, a significant decrease in off-flavor was observed in SS-F compared to the S-F both in dorsal and ventral muscle. This could be attribute to Lys/His, since they were flavor enhancers and masker for reducing the sensory defects caused by NaCl reduction in meat products.^19,22,48,49^ In addition, a significant increase in aroma was observed in SS-F compared to the S-F in ventral muscle. In Armenteros's study,^11^ it was shown that no significant differences were found with respect to aroma between the control (batch I, 100% NaCl) and batches II and III salted with substitutions up to 50% of NaCl by KCl. Therefore, the increase in aroma might be attribute to Lys/His. Lipolysis and oxidation constitute a important group of enzymatic reactions closely related to the final sensory quality, especially aroma of dry cured meat products. There was an initial breakdown of tri-acylglycerols and phospholipids by lipases and phospholipases, respectively, followed by oxidative reactions that produce aroma volatile compounds.^50^ The presence of Lys/His caused the increase of phospholipase activity, accelerating the hydrolysis of lipid, resulting in the increase of FAA, ultimately producing more aroma volatile compounds through oxidative reactions. The hypothesis was confirmed by correlation analysis (Table 11), where a positive correlation between aroma content and phospholipase activity was shown (*r* = 0.887, *p* < 0.05).

**Table 4:** The analysis of sensory evaluation in both dorsal and ventral muscle of salted fish with salt or SS treatments^a^

	Dorsal muscle		Ventral muscle	
	S-F	SS-F	S-F	SS-F
Intensity color	7.40 ± 1.08a	5.30 ± 0.82b	7.00 ± 1.16a	5.10 ± 1.20b
Saltiness	7.30 ± 0.50a	6.60 ± 0.16a	6.80 ± 0.32a	6.00 ± 0.49a
Aroma	7.10 ± 0.26a	7.00 ± 0.15a	6.50 ± 0.34b	7.00 ± 0.15a
Off-flavor	7.90 ± 0.88a	6.10 ± 0.37b	7.20 ± 0.23a	5.50 ± 0.07b
Overall acceptability	7.40 ± 0.27a	7.20 ± 0.92a	6.80 ± 0.47a	6.40 ± 0.43a

a Different lower-case letters in the same row indicate significant differences between salt and SS treatments in both dorsal and ventral muscle (*P* < 0.05).

Moreover, there was not significant difference in overall acceptability between S-F and SS-F, indicating that salt substitute containing Lys and His could been used to reduce NaCl without affecting the products flavor and acceptability.

### Comparison of free fatty acids

In the dorsal muscle, there are no any remarkable variations in the total content of polyunsaturated (PUFA), saturated (SFA) and monounsaturated fatty acids (MUFA) among the S-F and SS-F during the entire processing sequence, except SFA and PUFA at the end of 6 days. As shown in Table 5, a marked (*P* < 0.05) increase in the C15:0 and C18:3 content of SS-F was observed at the end of salting. On the 8^th^ day at the end of processing, the C15:0, C17:1, C18:3 and C20:3n3 content in SS-F was comparatively (*P* < 0.05) higher than that in S-F, indicating a trend toward lipolysis in SS-F. The increase of SFA (C15:0) might be due to the application of His in the salt substitute because His and iron ions constitute the catalytic center of the fatty acid desaturases, which were enzymes that could catalyze substrate SFA to synthesize UFA.^51^ Hence, it is suggested that the adding of histidine might cause a competitive binding to iron ions at the catalytic center, leading to a reduction in fatty acid desaturase activity, inhibiting the catalytic reaction, resulting in the increase of SFA. In parallel, the increase of MUFA (C17:1) and PUFA (C18:3 and C20:3n3) might be due to the increase of phospholipase activity (shown in the following analysis of lipase activities) caused by His/Lys, while a positve correlation between PUFA and phospholipase activity (*r* = 0.892, *p* < 0.05) was shown in Table 11.

**Table 5:** Free fatty acids composition (mg per g total lipid) in dorsal and ventral muscles of salted fish during processing^a^

		Processing steps								
		Raw	End of salting		4 days		6 days		8 days	
			S-F	SS-F	S-F	SS-F	S-F	SS-F	S-F	SS-F
Dorsal	C14:0	0.59 ± 0.101f	0.93 ± 0.034d	0.99 ± 0.102d	4.90 ± 0.302a	1.41 ± 0.003b	0.93 ± 0.005d	1.04 ± 0.021c	0.81 ± 0.002de	0.73 ± 0.101e
	C15:0	0.17 ± 0.004c	0.31 ± 0.012b	0.48 ± 0.021a	—	—	—	—	0.15 ± 001c	0.35 ± 0.021b
	C16:0	17.11 ± 1.032	18.56 ± 2.101	19.33 ± 3.011	16.58 ± 2.039	16.95 ± 2.102	18.16 ± 2.103	18.87 ± 2.012	18.47 ± 1.210	18.06 ± 1.023
	C17:0	0.26 ± 0.010c	0.21 ± 0.004c	0.25 ± 0.028c	—	—	0.56 ± 0.003a	—	0.14 ± 0.005cd	0.36 ± 0.002b
	C18:0	23.57 ± 2.210a	19.96 ± 4.120b	19.84 ± 3.198b	21.49 ± 3.218a	24.00 ± 3.021a	21.52 ± 2.103a	16.17 ± 1.091b	21.02 ± 2.104a	21.42 ± 2.102a
	C20:0	4.15 ± 0.321a	3.98 ± 0.278a	4.19 ± 0.701a	3.39 ± 0.410b	2.95 ± 0.087c	3.79 ± 0.210b	4.25 ± 0.012a	3.94 ± 0.201b	3.96 ± 0.218b
	C21:0	0.72 ± 0.042c	0.93 ± 0.005bc	0.97 ± 0.302bc	0.78 ± 0.012	1.15 ± 0.501b	0.73 ± 0.010c	1.96 ± 0.201a	0.82 ± 0.021c	0.82 ± 0.021c
	C22:0	1.01 ± 0.005	1.30 ± 0.006	1.09 ± 0.009	1.45 ± 0.003	1.09 ± 0.051	1.04 ± 0.002	—	1.12 ± 0.091	1.03 ± 0.021
	∑SFA	47.59 ± 4.125a	46.18 ± 3.491a	47.15 ± 4.411a	48.58 ± 5.102a	47.56 ± 4.811a	46.73 ± 3.121a	42.29 ± 2.911b	46.47 ± 4.252a	46.72 ± 3.501a
	C15:1	4.97 ± 1.067c	5.74 ± 1.001b	5.40 ± 1.201b	4.59 ± 0.109c	4.79 ± 0.304c	5.97 ± 1.001b	7.04 ± 1.004a	5.53 ± 0.0410b	5.55 ± 0.210b
	C16:1	—	—	—	—	—	—	—	0.17 ± 0.002	0.21 ± 0.001
	C17:1	0.23 ± 0.002b	0.24 ± 0.002b	0.35 ± 0.002a	—	—	—	—	0.27 ± 0.012b	0.33 ± 0.003a
	C18:1	33.89 ± 3.410	38.02 ± 5.121	37.36 ± 5.102	35.77 ± 4.910	37.98 ± 5.218	38.37 ± 4.210	36.63 ± 3.210	36.37 ± 3.410	36.70 ± 2.103
	C20:1	1.00 ± 0.002	—	—	1.12 ± 0.002	0.94 ± 0.004	0.83 ± 0.004	1.25 ± 0.003	0.86 ± 0.003	1.02 ± 0.003
	∑MUFA	40.09 ± 2.109b	44.00 ± 3.161a	43.11 ± 2.911a	41.48 ± 2.102ab	43.71 ± 2.161a	45.16 ± 1.902a	44.92 ± 4.211a	43.20 ± 3.171a	43.01 ± 1.821a
	C18:2	2.70 ± 0.071b	—	—	—	—	—	4.29 ± 0.219a	2.92 ± 0.013b	—
	C18:3	5.19 ± 1.560a	4.98 ± 0.217b	5.47 ± 1.021a	4.70 ± 0.089b	4.32 ± 0.791	4.19 ± 0.219bc	3.78 ± 0.501c	3.47 ± 0.510c	5.05 ± 0.502a
	C18:3N3	1.32 ± 0.061b	1.49 ± 0.008b	1.68 ± 0.002b	2.10 ± 0.002a	1.16 ± 0.004b	1.63 ± 0.013b	1.44 ± 0.230b	1.48 ± 0.031b	1.64 ± 0.401b
	C20:3	0.33 ± 0.001a	0.34 ± 0.001a	0.22 ± 0.009b	—	—	—	—	0.26 ± 0.005b	0.33 ± 0.002a
	C20:3N3	2.60 ± 0.059b	3.00 ± 0.171a	2.37 ± 0.301c	3.14 ± 0.208a	3.26 ± 0.081a	2.30 ± 0.029c	3.27 ± 0.081a	1.96 ± 0.105d	2.83 ± 0.204b
	C22:2	0.17 ± 0.005	—	—	—	—	—	—	—	—
	∑PUFA	12.33 ± 1.401a	9.81 ± 1.021b	9.74 ± 0.921b	9.93 ± 0.721b	8.73 ± 0.693bc	8.11 ± 0.712c	12.78 ± 1.302a	10.09 ± 0.802b	9.85 ± 0.791b
Ventral	C14:0	1.05 ± 0.219b	0.76 ± 0.235c	0.95 ± 0.130b	0.75 ± 0.020c	2.06 ± 0.219a	0.87 ± 0.129c	1.03 ± 0.021b	0.94 ± 0.103b	0.73 ± 0.219c
	C15:0	0.13 ± 0.001c		0.41 ± 0.021a	0.14 ± 0.006c		0.13 ± 0.003c	0.20 ± 0.008b	0.24 ± 0005b	0.26 ± 0.016b
	C16:0	17.39 ± 0.210	18.29 ± 0.034	17.96 ± 3.159	18.69 ± 2.104	16.02 ± 2.015	19.27 ± 1.230	19.42 ± 2.190	19.67 ± 1.210	18.91 ± 1.940
	C17:0	0.14 ± 0.103c	0.25 ± 0.004b	0.32 ± 0.034a	0.19 ± 0.006bc		0.21 ± 0.002b	0.27 ± 0.032b	0.21 ± 0.004b	0.16 ± 0.004c
	C18:0	22.42 ± 0.810a	20.02 ± 0.725a	22.01 ± 2.049a	20.58 ± 2.140a	21.10 ± 3.120a	19.53 ± 3.129b	15.75 ± 1.245c	20.15 ± 3.219a	21.36 ± 2.949a
	C20:0	2.88 ± 0.201c	3.91 ± 0.184b	3.39 ± 0.291b	4.64 ± 0.219a	3.41 ± 0.241b	4.43 ± 0.819a	4.46 ± 0.219a	4.18 ± 0.29a	4.19 ± 0.294a
	C21:0	0.66 ± 0.004c	1.06 ± 0.151a	0.77 ± 0.192b	0.86 ± 0.003	1.04 ± 0.034a	0.76 ± 0.003b	0.67 ± 0.023c	0.83 ± 0.005b	0.84 ± 0.019b
	C22:0	0.99 ± 0.029	0.81 ± 0.036	1.11 ± 0.023	1.18 ± 0.042	1.38 ± 0.024	1.08 ± 0.032	1.16 ± 0.210	1.10 ± 0.920	1.02 ± 0.004
	∑SFA	45.67 ± 4.219a	46.00 ± 4.325a	46.92 ± 4.489a	47.02 ± 4.311a	45.00 ± 2.192a	46.27 ± 4.219a	42.98 ± 2.811b	47.33 ± 3.981a	47.47 ± 4.219a
	C15:1	6.51 ± 1.201a	5.54 ± 0.404b	5.60 ± 0.912b	4.44 ± 0.491c	4.61 ± 0.304c	5.10 ± 0.590b	6.77 ± 0.319a	5.72 ± 0.892b	4.54 ± 0.409c
	C16:1				0.17 ± 0.001b		0.23 ± 0.039a	0.21 ± 0.003a	0.23 ± 0.034a	0.23 ± 0.005a
	C17:1	0.30 ± 0.003a	0.24 ± 0.007b		0.21 ± 0.005b		0.31 ± 0.004a	0.27 ± 0.021ab	0.27 ± 0.026ab	0.22 ± 0.009b
	C18:1	37.96 ± 3.091b	42.87 ± 2.567a	38.12 ± 3.102b	39.86 ± 4.120b	37.50 ± 4.210b	36.90 ± 4.219b	34.84 ± 3.219c	34.81 ± 4.219c	34.28 ± 2.918c
	C20:1	0.75 ± 0.029		0.81 ± 0.023	0.92 ± 0.003	1.18 ± 0.012	0.82 ± 0.023	1.33 ± 0.089	0.91 ± 0.029	1.08 ± 0.224
	∑MUFA	45.52 ± 3.191a	47.26 ± 4.179a	44.53 ± 3.971a	45.59 ± 4.012a	43.29 ± 2.987b	43.36 ± 4.211b	43.42 ± 4.271b	41.94 ± 2.911bc	40.36 ± 2.182c
	C18:2	2.88 ± 0.301b		2.87 ± 0.501b			2.94 ± 0.029b	3.80 ± 0.032a	2.88 ± 0.134b	2.78 ± 0.391b
	C18:3	3.12 ± 0.404bc	3.27 ± 0.011b	2.90 ± 0.023c	3.66 ± 0.401b	4.46 ± 0.123a	3.60 ± 0.219b	4.26 ± 0.421a	3.75 ± 0.341b	4.70 ± 0.502a
	C18:3N3	1.16 ± 0.102ab	0.78 ± 0.056b	1.33 ± 0.120ab	1.76 ± 0.301a	1.90 ± 0.021a	1.60 ± 0.109a	1.47 ± 0.201ab	1.55 ± 0.039ab	1.73 ± 0.104a
	C20:3	0.18 ± 0.009c	0.52 ± 0.035a				0.30 ± 0.004b	0.23 ± 0.009bc	0.23 ± 0.004bc	0.27 ± 0.008bc
	C20:3N3	1.46 ± 0.403d	1.67 ± 0.162d	1.46 ± 0.192d	1.98 ± 0.230cd	5.35 ± 0.812a	1.71 ± 0.205d	3.54 ± 0.219b	2.10 ± 0.024c	2.42 ± 0.230c
	∑PUFA	8.81 ± 0.921c	6.74 ± 0.391e	8.55 ± 0.818c	7.39 ± 0.719d	12.71 ± 1.029a	10.15 ± 1.002b	13.30 ± 1.321a	10.51 ± 0.928b	12.90 ± 0.192a

a Different lower-case letters in the same row indicate significant differences during the processing (*P* < 0.05).

In the ventral muscle, the content of C14:0 and C15:1 in SS-F was higher than that in S-F on the 6^th^ day, while a remarkable (*P* < 0.05) decrease was noticed in the final product. Among the PUFA, the C18:2, C18:3 and C20:3n3 contents in SS-F were significantly (*P* < 0.05) higher when compared to the S-F at the end of 6 days. During the entire process, the SS-F treatment had a remarkably higher content of total PUFA compared to the S-F (*P* < 0.05). PUFA is more susceptible to oxidative damage than MUFA and SFA,^52^ and the high PUFA content of ventral muscle in dry-salted fish makes it susceptible to oxidative degradation.

### Lipase activities between SS-F and S-F

Among the various types of lipolytic enzyme activities, highest activity was seen in neutral lipase during the processing from the 4 to 8 days of drying (Table 6). These results are in agreement with the findings of Zhou and Zhao,^53^ who previously reported the highest activity of neutral lipase in Jinhua ham. In dorsal muscle, no significant difference in neutral lipase activity was found between SS-F and S-F from 6 to 8 days, while a remarkably (*P* < 0.05) lower activity of acid lipase was found in SS-F compared to S-F. These findings are matched with the Zhang,^22^ who reported that the acid lipase in dry-cured loin was obviously inhibited by 0.05–0.4 M NaC/KCl and 0.05–0.25 M Lys. In the salt substitute of the present study, the NaCl and KCl content was up to 91%. Therefore, NaCl and KCl seemed to contribute a great inhibition in acid lipase effect. In parallel, the activity of phospholipase in SS-F was higher than S-F from beginning to 6 days, while no significant difference was found at the end of processing. In the Zhang's^22^ studies, they found that the phospholipase of dry-cured loin was not significantly influenced by partial replacement of NaCl by KCl, and the activity of phospholipase was increased in the presence of Lys/His. Therefore, the higher phospholipase activity was attributed to the presence of His/Lys. The results was in accordance with Liu's studies,^54^ who found that the phospholipase activity in dry cured beef was increased when treated with salt substitute containing Lys and His. This increased phospholipase activity in SS-F during the processing might be responsible for the increase of free fatty acid content described above.

**Table 6:** Lipase activities in dorsal and ventral muscles of salted fish during processing^a^

		Processing steps								
		Raw	End of salting		4 days		6 days		8 days	
			S-F	SS-F	S-F	SS-F	S-F	SS-F	S-F	SS-F
Dorsal	Neutral lipase	42.412 ± 3.279bcA	37.015 ± 0.661cdB	45.046 ± 0.744abA	47.360 ± 0.833aA	32.882 ± 1.311dB	39.387 ± 2.253bcdA	37.503 ± 0.232bcdA	36.502 ± 1.538cdA	37.257 ± 4.321bcdA
	Acid lipase	54.541 ± 1.344aA	40.232 ± 0.24bB	33.928 ± 0.609cB	37.655 ± 0.921bcA	26.462 ± 2.822deA	35.722 ± 0.797cA	23.659 ± 2.905eA	28.291 ± 3.031dA	16.485 ± 1.291fA
	Phospholipase	23.221 ± 0.494aA	19.962 ± 1.204bcdB	22.307 ± 2.229aA	18.391 ± 0.206cdA	21.853 ± 0.303abA	17.674 ± 2.695cdA	20.595 ± 1.218abcA	16.691 ± 1.808dA	18.201 ± 1.269bcdA
Ventral	Neutral lipase	39.496 ± 5.713bB	58.833 ± 4.441aA	39.322 ± 0.032bB	37.918 ± 1.448bB	37.705 ± 4.672bA	29.371 ± 2.155cdB	34.402 ± 0.763bcB	26.166 ± 1.019dB	31.847 ± 2.236bcdB
	Acid lipase	51.807 ± 3.617aB	48.799 ± 4.241abA	41.772 ± 4.601bA	32.325 ± 2.214cB	23.037 ± 1.421dB	19.031 ± 0.153deB	14.147 ± 1.476eB	15.206 ± 0.711deB	12.335 ± 1.391eB
	Phospholipase	23.585 ± 2.259bA	26.178 ± 2.206aA	21.714 ± 2.538abA	19.474 ± 0.556cdA	17.514 ± 1.653deB	13.051 ± 1.428fgB	15.615 ± 0.737efB	10.713 ± 0.916gB	14.919 ± 0.827efB

a Enzymatic activity was expressed as nmol of released 4-methylumbelliferone per h per g protein. Different capital letters in the same column indicate significant differences between the dorsal and ventral muscles for a given indicator (*P* < 0.05). Different lower-case letters in the same row indicate significant differences during the processing (*P* < 0.05).

In the ventral muscle, no significant difference in neutral lipase activity and acid lipase activity was found between SS-F and S-F, while the activity of phospholipase in the final SS-F was 39.26% higher than S-F (*P* < 0.05). Accordingly, a significant higher total PUFA content in SS-F was found as shown in Table 5. Kaneniwa^55^ found that the increase in free fatty acids was attributed to the hydrolysis of phospholipids in silver carp and noted that lipid hydrolysis was principally under the control of phospholipase. It is known that His is part of the active center of phospholipase in lysosomes^56^ and that higher phospholipase activity in porcine loin was obtained for 0.2–0.4 M Lys and 0.05–0.4 M His.^22^ Accordingly, our results shows that the application of His and Lys may affect the activity of lipase catalytic in dry-salted fish.

### Conjugated diene comparisons

Amount of conjugated diene was significantly (*P* < 0.05) decreased in SS-F group compared to the S-F in both dorsal and ventral muscles from 4 to 8 days of drying (Table 7). At the end of processing, the conjugated diene value in SS-F was 22.59% less than that in S-F for the dorsal muscle (*P* < 0.05). A similar phenomenon was found in the ventral muscle. It is generally accepted that PUFAs are particularly susceptible to hydrogen abstraction by free radical attack, becoming free radical intermediates themselves, which results in the arrangement of the double bond to conjugated dienes.^57^ Refsgaard^58^ also reported that the amino group of lysine can react with lipid free radicals or lipid peroxidation end-products. Therefore, it is suggested that in the present study the applications of Lys as salt substitute may decrease the chain reactions of reactive lipid free radicals. Moreover, in the final product, no considerable (*P* > 0.05) difference in conjugated diene was observed in SS-F or S-F between the ventral and dorsal muscle.

**Table 7:** Conjugated diene (%) in dorsal and ventral muscles of salted fish during processing^a^

Treatments	Processing steps				
	Raw	End of salting	4 days	6 days	8 days
**Dorsal**					
S-F	0.179 ± 0.018bA	0.213 ± 0.011bA	0.269 ± 0.003aA	0.262 ± 0.013aA	0.208 ± 0.026bAB
SS-F	0.179 ± 0.018bA	0.225 ± 0.007aA	0.209 ± 0.001aC	0.225 ± 0.004aB	0.161 ± 0.002bC

**Ventral**					
S-F	0.167 ± 0.039cA	0.218 ± 0.003abA	0.246 ± 0.003aB	0.222 ± 0.005cB	0.237 ± 0.002aA
SS-F	0.167 ± 0.039abA	0.237 ± 0.019bA	0.199 ± 0.001abD	0.190 ± 0.007abC	0.192 ± 0.001abBC

a Different capital letters in the same column indicate significant differences (*P* < 0.05). Different lower-case letters in the same row indicate significant differences during the processing (*P* < 0.05).

### Lipid oxidation

In the both muscles dorsal and ventral, no statistically (*P* > 0.05) variation was found in the amount of lipid among S-F and SS-F during processing (Table 8). In the ventral muscle, the amount of lipid was apparently higher than that in the dorsal muscle and the ventral muscle was more susceptible to oxidative damage due to its higher amount of polyunsaturated fatty acid.^59^ In our study, the TBARS value of the all samples of SS-F was remarkably (*P* < 0.05) lower than those of S-F throughout the processing. In the final SS-F product, the TBARS value was 41.51% and 54.29% less than that in the S-F product for dorsal muscle and ventral muscle, respectively. This might be due to the introduction of histidine into the salt substitute. Similarly, Zhai^60^ reported that the histidine, as a single oxygen scavenger, remarkably reduce the formation of MDA content and decrease the lipid peroxidation in myocardial membranes of rat.

**Table 8:** Lipid content (mg g^−1^) and lipid oxidation in dorsal and ventral muscles of salted fish during processing

		Processing steps								
		Raw	End of salting		4 days		6 days		8 days	
			S-F	SS-F	S-F	SS-F	S-F	SS-F	S-F	SS-F
Dorsal	Lipid content	5.39 ± 0.24aB	4.06 ± 0.9ab2bB	4.94 ± 0.12abB	4.69 ± 0.02abB	4.47 ± 0.15abB	4.79 ± 0.13abB	4.75 ± 0.26abB	4.62 ± 0.22abB	4.34 ± 0.41abB
	TBARS	0.017 ± 0.007gB	0.889 ± 0.031cB	0.277 ± 0.038fA	1.054 ± 0.004cB	0.651 ± 0.001eA	1.512 ± 0.008aB	1.122 ± 0.008bA	1.501 ± 0.007aB	0.878 ± 0.031dB
	Lipoxygenase	54.725 ± 5.126bB	72.167 ± 0.355aB	55.711 ± 1.635bB	56.898 ± 2.021bA	57.978 ± 0.364bA	55.295 ± 0.281bA	41.096 ± 0.455cB	54.462 ± 2.749bA	40.258 ± 0.638cB
Ventral	Lipid content	18.77 ± 0.98cA	26.18 ± 0.93aA	22.29 ± 0.12bA	14.73 ± 0.13dA	13.68 ± 1.34dA	21.36 ± 2.06bcA	22.86 ± 0.72bA	20.51 ± 1.37bcA	20.36 ± 1.03bcA
	TBARS	0.076 ± 0.015eA	1.214 ± 0.031cA	0.136 ± 0.008eB	1.724 ± 0.031bA	0.775 ± 0.054dA	2.704 ± 0.023aA	1.187 ± 0.023cA	2.715 ± 0.038aA	1.241 ± 0.008cA
	Lipoxygenase	82.666 ± 0.912bA	107.695 ± 10.355aA	95.448 ± 3.571aA	56.148 ± 2.198cA	56.408 ± 0.621cA	53.017 ± 2.075cA	46.136 ± 2.515dA	52.774 ± 4.648cA	45.501 ± 1.383dA

Lipoxygenase (LOX) was a non-heme ion containing dioxygenase that specially catalyzes the oxygenation of polyunsaturated fatty acids (PUFA) containing a *cis*, *cis*-1,4-pentadiene moiety (–CH=CH–CH_2_–CH=CH–) to produce conjugated unsaturated fatty acid hydroperoxides.^61^ Therefore, there was a negative correlation between PUFA content and LOX activity, which was confirm by the correlation analysis (*r* = −0.888, *p* < 0.05) in the present study (Table 11). As shown in Wang's study,^62^ the decrease of total free fatty acid as flavour precursors at the marinating stage may result from the promotion of oxidation by increasing LOX activity. LOX catalyzed the incorporation of dioxygen molecules into polyunsaturated fatty acid and was responsible for the initial post-mortem production of hydroperoxides in fish.^63^ After the end of 6 days of processing, SS-F presented a significant (*P* < 0.05) decrease in LOX activity compared with S-F in ventral muscle and dorsal muscle (Table 8). At the end of processing, the LOX activity in SS-F was 26.08% and 13.78% less than that in S-F for dorsal muscle and ventral muscle, respectively. Ling^64^ stated that the availability of lysine caused higher inhibitory effect of paederosidic acid on the LOX activity, furthermore, the LOX activity inhibition was shown only with the presence of lysine in the case of mixtures of gardenogenins A and B. Consequently, it is suggested that the lysine in the salt substitute have a role in the inhibition of LOX activity in dry-salted fish.

### Protein carbonyl measurements

The carbonyl contents in dorsal and ventral muscles of salted fish during processing were shown in Table 9. The amount of carbonyl groups significantly increased (*p* < 0.05) in both dorsal and ventral muscles during processing. In the dorsal muscle, no significant difference (*p* > 0.05) was found in the amount of carbonyl content between S-F and SS-F. However, carbonyl contents in S-F was higher than that in SS-F from 4 to 8 days of drying for the ventral muscle (*p* < 0.05), indicating that the His and/or Lys might inhibit the protein oxidation. The result was similar with the study by Xu,^65^ who found that Lys- or Arg-treated sausages had significantly (*p* < 0.05) lower carbonyl values than the control during 10 to 25 day of storage. Many studies had revealed that positive correlation between protein oxidation and lipid oxidation.^66,67^ In the present study, a positive correlation between protein oxidation and lipid oxidation was also shown in Table 11, and the correlation coefficient between carbonyl contents and TBARS was 0.964 (*p* < 0.01). Amino acids with NH or NH_2_ moiety on their side chains can react with free oxygenated radical from lipolysis, then transforming into carbonyl groups. Carbonyl groups may be introduced into proteins by reactions with aldehydes (4-hydroxy-2-nonenal, malondialdehyde) produced during lipid peroxidation.^68^ In the previous study, Lys was reported to have metal chelating activity,^69^ indicating that Lys could prevent the oxidation of proteins. In our study, it was found that salt substitute inhibited the lipid oxidation due to the presence of histidine, which was a single oxygen scavenger.^60^

**Table 9:** Carbonyl content (nmol per mg protein) in dorsal and ventral muscles of salted fish during processing^a^

Treatments	Processing steps				
	Raw	End of salting	4 days	6 days	8 days
**Dorsal**					
S-F	1.979 ± 0.063cB	2.023 ± 0.106cB	2.405 ± 0.190bC	2.645 ± 0.210abC	3.362 ± 0.403aB
SS-F	1.979 ± 0.063cB	2.156 ± 0.101bcB	2.851 ± 0.281abC	2.249 ± 0.100bC	3.217 ± 0.251aB

**Ventral**					
S-F	3.017 ± 0.100eA	3.954 ± 0.087dA	5.134 ± 0.025cA	7.197 ± 0.532bA	9.370 ± 0.253aA
SS-F	3.017 ± 0.100dA	4.077 ± 0.057cA	4.668 ± 0.360cB	6.156 ± 0.251bB	8.641 ± 0.160aB

a Different capital letters in the same column indicate significant differences (*P* < 0.05). Different lower-case letters in the same row indicate significant differences during the processing (*P* < 0.05).

### Sulphydryl content

**Fig. 1:**
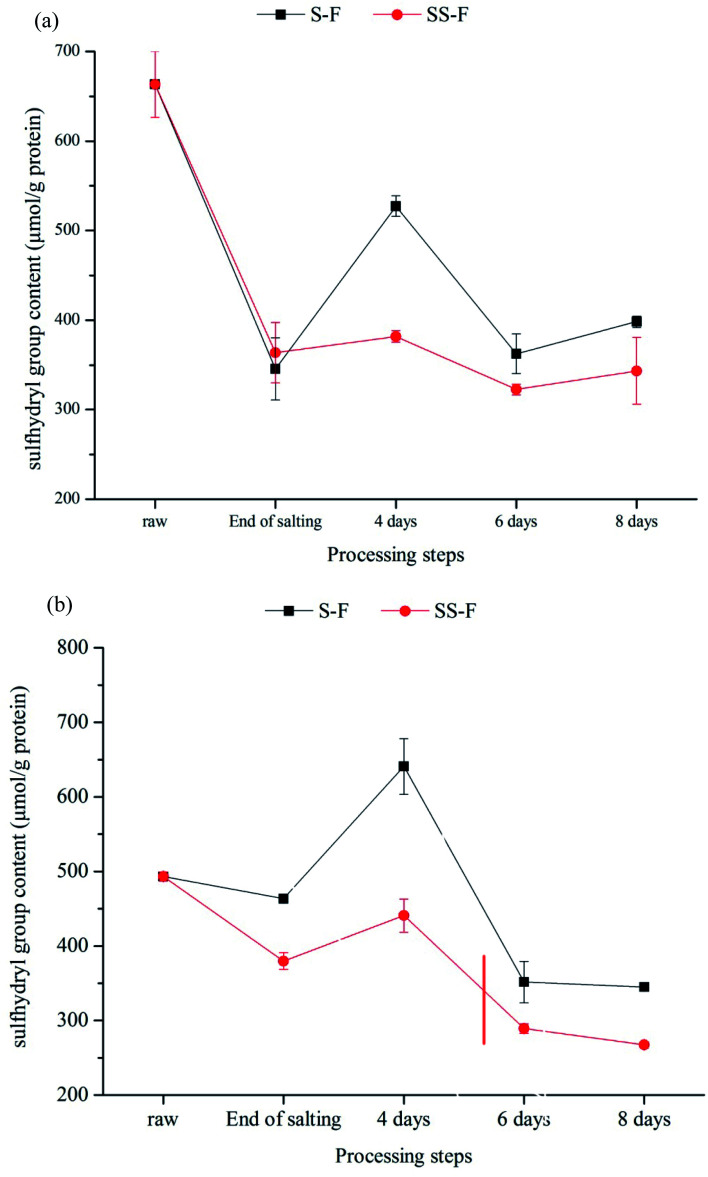
Changes in sulfhydryl group content in dorsal (a) and ventral muscles (b) of salted fish with salt or SS treatments during processing.

Protein oxidation is also associated with a decrease in sulphydryl groups, which are converted into disulphide.^70^ The level of SH was shown in Fig. 1. There was a significant changes in SH content during processing (*p* < 0.05). In general, the SH content was decreased as processing time proceeding, which might correspond to the oxidation of accessible free thiol groups from cysteine residues located at the protein surface.^71^ There was a increase in SH content from end of salting days to 4 days both in the dorsal and ventral muscle, due to the breakage of disulfide bond caused by proteolysis. In the dorsal muscle, compared with SH content in S-F, the SH content in SS-F decreased by 27.61%, 11.01% and 13.77% from 4 days to 8 days, respectively. Meanwhile, it was decreased by 31.19%, 17.74% and 22.51% in the ventral muscle, respectively. The result indicated that the introduction of Lys and/or His increased the loss of free thiol groups. Zhang^72^ found that sage also accelerated the loss of thiol although it prevented protein oxidation. In Xu's^65^ study, he found that Lys treated sausages showed significantly lower (*p* < 0.05) thiol levels than those in the control during storage. Liu^54^ also reported that Lys and His increased the loss of thiol groups. In the presence of positively charged Lys and His, the α,β-unsaturated compounds reacted with thiols, ultimately contributing to the decrease in thiol levels.

### Enzyme activities

**Fig. 2:**
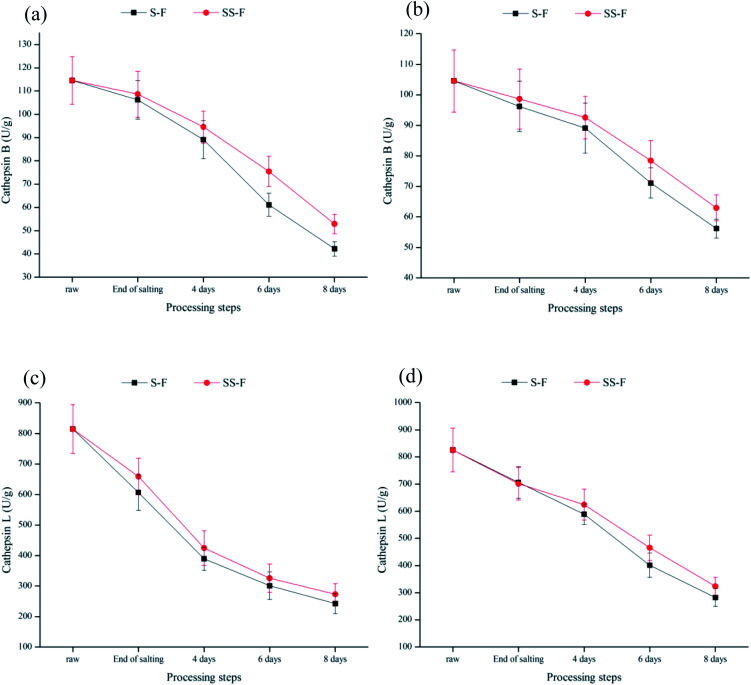
Changes in relative activity of cathepsins B and L in dorsal (a and b) and ventral muscles (c and d) of salted fish with salt or SS treatments during processing.

Cathepsins B and L were lysosomal proteinases played an important role in the proteolytic chain taking place during the curing process and affect the development of the sensory characteristics of meat products.^73–76^ The activities of cathepsins B and L were significantly decreased (*p* < 0.05) both in the dorsal and ventral muscles during processing (Fig. 2), which was primarily because of the inhibition effect of salt on their activities.^76^ In the dorsal muscle, compared with S-F, cathepsin B activity in SS-F was increased by 2.30%, 6.13%, 23.47% and 25.50% from end of salting days to 8 days, respectively; while cathepsin L activity in SS-F was increased by 8.65%, 9.11%, 8.08% and 12.70% from end of salting days to 8 days, respectively. Same tendency was observed in the ventral muscles. The results illustrated that salt substitute increased enzyme activity. Armenteros^77^ found that KCl had a similar inhibition effect to NaCl for cathepsin B and L. Therefore, the higher activity of cathepsin B and L was due to the replacement of NaCl by Lys and His.

### FAA content

The FAA played an important role in the development of flavor.^78^ Therefore, it was important to determine their release in dry-cured fish. Changes of FAA content in dorsal and ventral muscles of salted fish treated with NaCl and salt substitute during processing were shown in Table 10. In the present study, the 17 FAA content at the end of processing was used as a measure of proteolytic activity. Statistical analysis displayed that total FAA was significantly (*P* < 0.05) increased throughout the whole manufacture process both in dorsal and ventral muscle, and the principal increase was in Gly, lys, Glu and Ala. This was in accordance with the previous studies.^79–81^ In addition, total FAA in SS-F was higher than that in S-F, which was increased by 15.40%, 17.43%, 15.28% and 9.88% in dorsal muscle, and 33.28%, 7.35%, 45.07% and 16.19% in ventral muscle, from end of salting to 8 days, respectively. In the dorsal muscle, at the end of processing, the content of Glu and Lys in SS-F was higher than that in S-F, while Cys, Met and Phe was found to be lower than that in S-F (*p* < 0.05). In the ventral muscle, the content of Ala, Cys and lys in SS-F was higher than hat in S-F, while Asp was found to be lower than that in S-F (*p* < 0.05). These results indicated that salt substitute increased the preteolysis, while total FAA content showed a positive correlation (*r* = 0.890, *p* < 0.05) with cathepsin B activity (Table 11). Armenteros^77^ found that the replacement of NaCl by KCl didn't cause significant changes in total FAA content. Hence, these results indicated that the introduction of His and/or Lys caused the release of FAA through proteolysis of proteins by endogenous enzymes and aminopeptidases activity.^82,83^

**Table 10:** Changes in free amino acids (mg/100 g) in dorsal and ventral muscles of salted fish with salt or SS treatments during processing^a^

FAA		Processing steps								
		Raw	End of salting		4 days		6 days		8 days	
			S-F	SS-F	S-F	SS-F	S-F	SS-F	S-F	SS-F
Dorsal	Asp	0.67 ± 0.02e	1.04 ± 0.13d	0.80 ± 0.04e	1.75 ± 0.26c	1.21 ± 0.12d	2.60 ± 0.17b	1.57 ± 0.11c	3.23 ± 0.28a	1.98 ± 0.31c
	Thr	ND	ND	ND	ND	ND	ND	ND	ND	ND
	Ser	5.59 ± 0.89e	5.64 ± 0.74e	5.78 ± 0.29e	7.44 ± 0.91d	8.46 ± 0.59cd	9.20 ± 1.01c	11.59 ± 1.23b	12.83 ± 1.20ab	14.94 ± 1.45a
	Glu	2.38 ± 0.49g	2.90 ± 0.04g	5.43 ± 0.02f	7.78 ± 0.74e	8.04 ± 0.29e	10.60 ± 0.92d	13.13 ± 1.21c	19.38 ± 2.31b	28.86 ± 2.19a
	Gly	37.97 ± 2.89e	50.45 ± 4.29cd	46.34 ± 4.21d	52.78 ± 3.29c	60.58 ± 5.21bc	54.97 ± 3.21c	66.60 ± 4.21b	83.62 ± 4.98a	85.85 ± 9.21a
	Ala	19.85 ± 3.19c	18.99 ± 2.17c	32.53 ± 1.54a	18.62 ± 1.82c	31.15 ± 2.21a	23.66 ± 2.98b	33.81 ± 2.13a	34.11 ± 1.89a	38.34 ± 2.19a
	Cys	0.80 ± 0.04a	0.85 ± 0.004a	0.54 ± 0.05b	0.87 ± 0.04a	0.63 ± 0.04ab	0.82 ± 0.03a	0.58 ± 0.02b	0.86 ± 0.03a	0.48 ± 0.09b
	Val	4.56 ± 0.21c	4.41 ± 0.42c	4.15 ± 0.53c	6.09 ± 0.19b	6.45 ± 0.34b	7.92 ± 0.69b	6.01 ± 0.54b	12.94 ± 0.59a	12.60 ± 1.10a
	Met	2.01 ± 0.09d	2.12 ± 0.04d	2.01 ± 0.09d	2.99 ± 0.04c	3.08 ± 0.21c	4.28 ± 0.40b	4.64 ± 0.29b	7.73 ± 0.12a	4.48 ± 0.49b
	Ile	3.51 ± 0.39c	3.79 ± 0.73c	3.74 ± 0.21c	4.90 ± 0.08bc	5.66 ± 0.23b	6.12 ± 0.49b	5.36 ± 0.33b	9.58 ± 0.23ab	11.77 ± 1.23a
	Leu	6.27 ± 0.83d	6.15 ± 0.92d	5.69 ± 0.79d	8.13 ± 0.89c	8.73 ± 1.21c	10.01 ± 1.01b	10.22 ± 0.89b	16.84 ± 1.23a	17.89 ± 1.89a
	Tyr	5.45 ± 0.39d	5.00 ± 0.49d	4.19 ± 0.92de	6.83 ± 0.49cd	5.92 ± 0.97cd	7.49 ± 0.94b	8.12 ± 0.52b	13.35 ± 1.45a	15.57 ± 1.69a
	Phe	3.98 ± 0.25f	4.00 ± 0.56ef	3.99 ± 0.45f	5.25 ± 0.53e	5.64 ± 0.29e	7.04 ± 0.19c	6.53 ± 0.14d	12.42 ± 1.12a	8.89 ± 0.21b
	Lys	15.47 ± 1.59c	15.65 ± 2.09c	58.80 ± 7.39a	20.49 ± 2.12b	53.12 ± 8.21ab	20.71 ± 1.97b	59.53 ± 4.12a	29.93 ± 2.89b	64.25 ± 3.21a
	His	116.32 ± 5.21b	115.92 ± 3.92b	108.07 ± 5.20b	116.98 ± 4.90b	110.81 ± 4.39b	131.55 ± 5.39a	122.34 ± 12.81a	131.60 ± 10.39a	128.89 ± 10.21a
	Arg	4.63 ± 0.21c	5.90 ± 0.21b	5.32 ± 0.49b	6.74 ± 0.79a	7.56 ± 0.29a	7.52 ± 0.49a	7.39 ± 0.39a	7.43 ± 0.82a	7.98 ± 0.29a
	Pro	26.48 ± 4.19ab	25.63 ± 2.19ab	22.00 ± 2.03b	23.28 ± 2.94b	23.73 ± 3.04b	30.97 ± 4.21a	27.87 ± 1.29ab	31.62 ± 3.21a	25.37 ± 1.21ab
	Total	255.27 ± 10.21c	267.4 ± 11.02c	308.58 ± 12.39b	289.17 ± 12.21c	339.56 ± 13.89b	332.86 ± 12.31b	383.72 ± 15.21b	424.24 ± 14.21b	466.16 ± 19.29a
Ventral	Asp	0.92 ± 0.02e	1.33 ± 0.31d	1.10 ± 0.21d	2.75 ± 0.41cd	2.38 ± 0.49cd	3.71 ± 0.05c	1.39 ± 0.06d	8.66 ± 0.61a	6.71 ± 0.55b
	Thr	ND	ND	ND	ND	ND	ND	ND	26.60 ± 1.36a	24.01 ± 1.13a
	Ser	4.91 ± 0.43c	4.96 ± 0.68c	4.96 ± 0.26c	8.49 ± 0.92b	8.42 ± 0.59b	10.11 ± 1.02b	9.53 ± 1.42b	15.50 ± 0.36a	12.73 ± 1.02ab
	Glu	2.26 ± 0.09f	4.70 ± 0.41e	4.92 ± 0.31e	11.57 ± 1.52d	12.62 ± 2.13cd	14.08 ± 2.15c	25.06 ± 2.24b	31.44 ± 2.53a	29.79 ± 2.16a
	Gly	25.49 ± 1.05d	43.21 ± 3.21c	49.23 ± 4.15c	44.30 ± 4.21c	42.33 ± 3.21c	49.11 ± 3.21c	63.81 ± 4.16b	78.84 ± 6.21a	75.47 ± 4.62a
	Ala	16.87 ± 0.93de	9.81 ± 1.04e	53.70 ± 3.29b	21.20 ± 2.18d	36.60 ± 2.41c	23.74 ± 1.49d	95.75 ± 6.29a	41.14 ± 2.59bc	94.91 ± 5.21a
	Cys	0.80 ± 0.04c	0.67 ± 0.04d	0.53 ± 0.05e	0.98 ± 0.04b	0.70 ± 0.04d	0.63 ± 0.05de	0.80 ± 0.17c	0.94 ± 0.04b	2.78 ± 0.59a
	Val	4.26 ± 0.21e	5.03 ± 0.56d	4.21 ± 0.31e	7.29 ± 0.93c	7.69 ± 0.62c	9.27 ± 0.91b	14.49 ± 2.19a	14.92 ± 2.16a	13.69 ± 1.32a
	Met	2.07 ± 0.19d	2.43 ± 0.21d	2.99 ± 0.05d	3.35 ± 0.03c	3.58 ± 0.41c	5.03 ± 0.49b	5.26 ± 0.59b	8.71 ± 0.52a	7.84 ± 0.59a
	Ile	3.32 ± 0.29e	4.35 ± 0.35d	3.73 ± 0.41d	5.89 ± 0.41c	6.75 ± 0.51b	7.21 ± 0.21b	10.68 ± 1.06a	11.01 ± 1.05a	13.11 ± 1.32a
	Leu	5.91 ± 0.69cd	7.02 ± 0.59c	5.67 ± 0.69d	9.95 ± 0.74bc	10.42 ± 1.21b	11.96 ± 0.59b	18.11 ± 1.42a	19.38 ± 1.52a	19.57 ± 1.67a
	Tyr	5.34 ± 0.54d	5.88 ± 1.03d	4.39 ± 0.42d	8.59 ± 0.59b	7.30 ± 0.59c	9.14 ± 0.25b	14.15 ± 1.56a	15.58 ± 1.32a	15.82 ± 1.24a
	Phe	3.81 ± 0.25e	4.65 ± 0.54de	4.17 ± 0.51de	6.71 ± 0.39c	6.71 ± 0.48c	8.58 ± 0.49b	8.09 ± 0.79b	14.44 ± 1.03a	13.93 ± 1.31a
	Lys	13.81 ± 1.59e	18.08 ± 1.04e	90.22 ± 3.21a	24.84 ± 2.14de	57.86 ± 2.17c	24.31 ± 1.54de	80.31 ± 5.29ab	35.40 ± 2.54d	68.93 ± 5.24b
	His	113.01 ± 8.21d	130.36 ± 10.32b	111.57 ± 4.63d	132.57 ± 10.21b	120.41 ± 5.14cd	136.70 ± 12.14ab	131.39 ± 9.25b	159.64 ± 10.21a	153.23 ± 10.42a
	Arg	4.31 ± 0.49d	6.57 ± 0.58c	5.06 ± 0.41cd	8.43 ± 0.59b	7.95 ± 0.18b	8.02 ± 0.69b	8.10 ± 0.61b	9.90 ± 0.29a	10.78 ± 1.03a
	Pro	26.88 ± 2.19b	28.36 ± 2.85b	23.32 ± 2.13b	38.63 ± 2.15a	28.49 ± 2.84b	35.79 ± 2.26a	31.54 ± 1.53ab	39.47 ± 1.23a	54.35 ± 3.69a
	Total	233.95 ± 10.21d	277.44 ± 11.29d	369.77 ± 13.29c	335.55 ± 13.21c	360.21 ± 12.59c	357.38 ± 12.54c	518.46 ± 18.21b	531.57 ± 13.56b	617.63 ± 19.29a

a ND means not detected. Different lower-case letters in the same row indicate significant differences during the processing (*P* < 0.05).

**Table 11:** Correlation among physicochemical parameters, sensory properties, lipolysis, lipid oxidation, proteolysis and protein oxidation during dry-salted grass carp processing^a^

	Moisture	L*	Hardness	Aroma	PUFA	Neutral lipase	Acid lipase	Phospholipase	TBARS	Lipoxygenase	Cathepsin B	FAA	Carbonyl content
Moisture	1												
L*	−0.995**	1											
Hardness	−0.843*	0.890*	1										
Aroma	0.003	−0.065	−0.147	1									
PUFA	−0.945**	0.954**	0.923**	0.153	1								
Neutral lipase	0.719	−0.675	−0.295	0.218	0.457	1							
Acid lipase	0.901*	−0.859*	−0.523	−0.017	0.730	0.930**	1						
Phospholipase	0.894*	−0.856*	−0.608*	0.887*	0.892*	0.729	0.902*	1					
TBARS	−0.996**	0.995**	0.845*	−0.246	0.924**	−0.744	−0.902*	−0.873*	1				
Lipoxygenase	−0.944**	0.969**	0.914*	0.298	−0.888*	−0.647	−0.782	−0.740	0.964**	1			
Cathepsin B	−0.438	0.499	0.899*	0.137	0.690	0.302	−0.011	−0.236	0.411	0.501	1		
FAA	−0.997**	0.992**	0.857*	0.062	0.967**	−0.670	−0.877*	−0.896*	0.987*	0.929**	0.890*	1	
Carbonyl content	−0.971*	0.985**	0.942**	0.023	0.987**	−0.539	−0.771	−0.817*	0.964**	0.951**	0.638	0.980	1

a * means *p* < 0.05; **means *p* < 0.01.

## Conclusions

In summary, salt substitute containing Lys and His accelerated the hydrolysis of protein and lipid and inhibit the oxidation of protein and lipid in dry-salted grass carp during processing. Salt substitute induced a marked reduction (>50%) in the Na content and enhanced the phospholipase activity in dry-salted fish compared to the NaCl treated samples. Simultaneously, slightly higher lipolysis was observed in the fish samples treated with the salt substitute. In addition, the TBARS value, LOX activity and the value of conjugated diene were lower in the both muscles of SS-F group than S-F. The addition of Lys and His increased the activity of cathepsin B and L, leading to the increment of FAA content, while decreased the content of protein carbonyls and thiol groups. The use of salt substitute decreased the color, off-flavor and hardness of fish samples accompanied by the increase of aroma, without affecting overall acceptability of final products, indicating that NaCl could totally replaced by salt substitute containing Lys and His during the processing of the dry-salted grass carp. Therefore, it was suggested that the partial replacement of NaCl by Lys and His might be a possible approach to reduce the amount of sodium in dry-cured meat products, although its effect on the physico-chemical and sensory properties of different dry-cured meat products remained to be further studied.

## Conflicts of interest

The authors declare no conflict of interest.

## Supplementary Material
